# Risking infanticide or losing precious time? Delay of reproduction in a short-lived mammal

**DOI:** 10.1093/beheco/araf041

**Published:** 2025-05-03

**Authors:** Lea Vodjerek, Jasmin Firozpoor, Heiko G Rödel, Jana A Eccard

**Affiliations:** Animal Ecology, Institute for Biochemistry and Biology, University of Potsdam, Maulbeerallee 1, 14469 Potsdam, Germany; Animal Ecology, Institute for Biochemistry and Biology, University of Potsdam, Maulbeerallee 1, 14469 Potsdam, Germany; Laboratoire d’Ethologie Expérimentale et Comparée (LEEC) UR 4443, Université Sorbonne Paris Nord, 99 Av. Jean Baptiste Clément, 93430 Villetaneuse, France; Animal Ecology, Institute for Biochemistry and Biology, University of Potsdam, Maulbeerallee 1, 14469 Potsdam, Germany; Berlin-Brandenburg Institute of Advanced Biodiversity Research (BBIB), Altensteinstraße 34, 14195 Berlin, Germany

**Keywords:** infanticide, pregnancy replacement, sexual conflict, *Myodes glareolus*

## Abstract

Nonparental infanticide, or killing of conspecific young infants, is an extreme form of sexual conflict that is widespread throughout the animal kingdom. One of the female counterstrategies to reduce the damage of infanticide is the “Bruce effect,” ie the termination of a pregnancy sired by the former breeding male after the invasion of a new breeding male. Using bank voles (*Myodes glareolus*) as experimental subjects we phenotyped males for infanticidal types (attacking unrelated pups or not), and we exchanged the breeding male in the early second trimester of a potential pregnancy, allowing the female to terminate the pregnancy and breed with the new male (pregnancy replacement). We found that the proportion of late litter was significantly higher if the second male encountered was infanticidal, and males’ probability to reproduce was affected by both infanticidal tendency and the sequence in which it was presented to the female. We found no connection between infanticidal tendencies and male quality, and females’ choice was not affected by male quality or female parity. Consequently, the infanticidal status of the males and the sequence in which the males are presented may trigger females to exchange a pregnancy and reproduce with an infanticidal male. Thus, the functional Bruce effect may be considered as a form of sequential mate choice.

## Teaser

The primary objective of this project is to assess whether males’ infanticidal tendencies influence female reproductive choice in small mammals. We focused on delay in reproduction when the second male was infanticidal. We found that late pregnancies were more likely if the second male was infanticidal, showing that females would possibly terminate their first pregnancies as a counterstrategy to avoid infanticide by males, even at the cost of delaying reproduction.

## Introduction

Sexual conflict is present in many species, often due to the sexual dimorphism in gamete size and mating costs (Trivers 1972; Parker 1979). Furthermore, in most species, there is a difference in parental investment, where males have a higher reproductive potential than females (Trivers 1972; Clutton-Brock and Parker 1992), as is the case in mammals. Conflict requires interactions between males’ and females’ reproductive interests, and one example of such conflict is infanticide by non-parental males, where females lose their reproductive investment, and males increase the probability of mating with the female (Agrell et al. 1998; Ebensperger 1998; Arnqvist 2004). Nonparental infanticide, or killing of young infants by conspecifics is widespread throughout the animal kingdom, from invertebrates to vertebrates (Hausfater and Hrdy 1984; Elgar and Crespi 1992; Parmigiani and Vom Saal 1994). Several hypotheses have been proposed to explain the occurrence of nonparental infanticide by males (Agrell et al. 1998; Ebensperger 1998), eg to get access to better food or nesting site for the perpetrator or its’ offspring (“the resource competition hypothesis”; Rudran 1973; Hrdy 1979). The “sexual selection hypothesis” suggests that males commit infanticide in order to shorten the inter-birth interval and increase their chance of mating with the female (Hrdy 1979; Ebensperger 1998). Originally, infanticide was viewed as maladaptive and pathological conduct (Calhoun 1962) or a behavior aimed at regulating population growth for the benefit of the species. However, further understanding acknowledges infanticide as an adaptive behavioral strategy used to enhance the individual fitness of the perpetrator (Hrdy 1979; Hausfater and Hrdy 1984; Huck 1984; Pierotti 1991).

Infanticidal behavior has primarily evolved in group-living species in which a few dominant males monopolize the reproduction (Lukas and Huchard 2014), and where the care for the offspring would prevent females from mating again, at least until the current offspring is independent. However, in species with post-partum estrus (ie most rodents) there are other benefits of infanticide occurrence since females can be both lactating and pregnant at the same time, one of them being possibly the larger size of the next litter (Elwood and Kennedy 1990). In mammalian species, infanticide has been recorded under both laboratory and wild conditions in over 100 species, mostly primates, carnivores and diurnal rodents (Hausfater and Hrdy 1984; Hoogland 1985; Parmigiani and Vom Saal 1994; Harestad and Davis 1996; Ebensperger 1998; Ebensperger and Blumstein 2007; Lukas and Huchard 2014). As nonparental infanticide reduces the overall fitness of the parents (Chapman and Hausfater 1979), it is expected that species evolve strategies preventing possible infanticide (Chapman and Hausfater 1979; Glass et al. 1985; Hiraiwa-Hasegawa 1988).

Pregnancy termination (before implantation), also known as the ‘Bruce effect’ (Bruce 1959, 1960; Milligan 1976), is currently understood as one of the female counterstrategies to male infanticide. Moreover, it might reduce a female’s investment into offspring that might subsequently be killed by the invading, non-sire male (Hrdy 1979; Schwagmeyer 1979; Huck 1984; Ebensperger 1998; Marashi and Rülicke 2012; Bartoš et al. 2022). This hypothesis predicts (1) a direct correlation between the rate of pregnancy termination and the risk of infanticide, and (2) that under similar conditions of infanticidal risk, females who interrupt their pregnancies should wean more offspring than females who retain their former pregnancies (Ebensperger 1998). In mice, eg it has been shown that females can block pregnancy before implantation (Bruce 1959, 1960); those that do not block their pregnancies have a higher chance of losing entire litters in response to an infanticidal perpetrator compared to their counterparts (Elwood and Kennedy 1990). Still, different species of voles and rats can obstruct pregnancies at later stages, ie after implantation (Schwagmeyer 1979; Heske and Nelson 1984; Heske 1987; Storey and Snow 1990; Marashi and Rülicke 2012). In its original form, the Bruce effect refers to a pregnancy block that would happen before implantation, ie mechanistical definition of Bruce effect (Bruce 1959, 1960). We, on the other hand, follow the functional definition of Bruce effect, that states: “when females terminate pregnancies after some form of sensory exposure (olfactory, visual, auditory, or tactile) to nonsire males” (Zipple 2020; Zipple et al. 2021). This definition would cover all pregnancy failures (Roberts et al. 2012), both before, and after implantation, such as we will discuss in this experiment.

For individuals of short-lived species, often with annual density fluctuations, it is important to reproduce as early as possible to contribute to the future population. Thus, by interrupting a pregnancy at late stages, females lose valuable time and possibly a major proportion of fitness (Eccard et al. 2017). In several species of rodents, evidence suggests there is a positive association between the risk of infanticide and the occurrence of a pregnancy block as a great proportion of rodent adult and subadult males are infanticidal (Blumstein 2000). Numerous studies have proposed that engaging in copulation alone is sufficient for a female to inhibit infanticide by that male (Elwood 1977; Vom Saal and Howard 1982; Palanza and Parmigiani 1991), whereas others argue that in addition to mating, physical contact or proximity to the female or her nest is necessary for the inhibition of infanticide in male rodents (Huck et al. 1982; McCarthy and Vom Saal 1986; Mennella and Moltz 1988; Cicirello and Wolff 1990).

In this paper, we investigated the predictive power of the infanticidal status of males on the timing of pregnancies and whether there is a link between male quality and infanticidal status. Furthermore, we investigated whether infanticidal status was repeatable over long periods of life. We paired females successively with two previously phenotyped males and assumed that females could choose among potential sires in order to maximize their fitness. In this experiment we reviewed whether females might use pregnancy termination and remating (ie pregnancy replacement) to avoid potential infanticide by males(Eccard et al. 2017; Vodjerek et al. 2024). We specifically predict, that if females based their decision on infanticide risk, and one of the males is infanticidal, the offspring will be sired by this male. If both males are infanticidal, the offspring will be sired by the last male paired with the female, since females have a higher chance to wean the offspring sired by the last male they encounter, otherwise, the offspring of the previous male might be killed by the new male. We expected the infanticidal status of the males and the sequence in which the males were presented to the females to affect the timing of the births and the males’ probability of reproducing. Therefore, if we consider late litters a form of infanticide avoidance, potentially through pregnancy replacement (functional Bruce effect), we would expect a higher proportion of late litters in the sequences where the second male was infanticidal compared to sequences where the second male was non-infanticidal. Further, we investigated alternative explanations affecting female mate choice including male quality and female parity. Lastly we investigated whether infanticide risk changed over time relative to the age-dependent vulnerability of the offspring (Elwood 1977; Labov 1980; Cicirello and Wolff 1990; Vihervaara et al. 2010).

## Methods

### Study species and animal-keeping

The breeding system of bank voles is promiscuous (Klemme et al. 2007; Klemme and Ylönen 2010), and the percentage of multiple sired litters was shown to be between 20% and 30% in wild populations (Ratkiewicz and Borkowska 2000). Males do not provide any care for their offspring (Ylönen et al. 2017). In bank voles, both females and males were shown to be infanticidal (Ylönen 1997; Agrell et al. 1998; Vihervaara et al. 2010; Opperbeck et al. 2012), and the ratio of infanticidal to non-infanticidal males within the population can differ. The infanticidal tendency of a male is reported to be highly repeatable within the individual, and also heritable (Poikonen et al. 2008; Korpela et al. 2010; Mappes et al. 2012), but body size and dominance were not related to infanticidal behavior (Horne and Ylönen 2002; Korpela et al. 2010).The threat for the offspring is highest when pups are less than 10 d old (Horne and Ylönen 2002; Korpela et al. 2010). Furthermore, a recent study has shown that females are able to assess the threat of infanticide by using male odors and they adjust their behavior accordingly (Breedveld et al. 2019), and that males are less likely to kill their offspring due to familiarization with the female (Korpela et al. 2010).

Like many other rodent species, bank voles are seasonal breeders with females becoming receptive multiple times throughout the season (Andersson and Gustafsson 1982). Females are receptive either during post-partum estrus (after parturition) or cyclic estrus, with the former being the most common mode of reproduction (Klemme et al. 2011). Cyclic estrus can be triggered by male presence or odor, and mostly occurs if females have not mated yet or if the previous mating has been unsuccessful (Dewsbury 1990; Klemme et al. 2011). Gestation lasts between 18 and 20 d for females that mated during cyclic estrus and litter size usually ranges between 4 and 7 pups (Gustafsson et al. 1980; Bujalska 1990).

Experiments were conducted in the housing facility of Animal Ecology Potsdam in the spring and summer of 2022, 2023 and 2024. We used either wild-captured bank voles, or laboratory-born F1 and F2 descendants of the wild captured individuals. All animals were housed singly immediately if they were trapped in the wild, or after weaning if they were born in the laboratory. All individuals were adults, overwintered (ie born in the previous season) or year-born (ie sexually matured individuals born in the season). Females used in the experiment could have been either nulliparous (first time breeders) or parous (already was breeding and produced at least one litter). All males and females were reproductively active, females were not pregnant, and both males and females were weighed before the experiment.

### Experimental setup

**Fig. 1.**
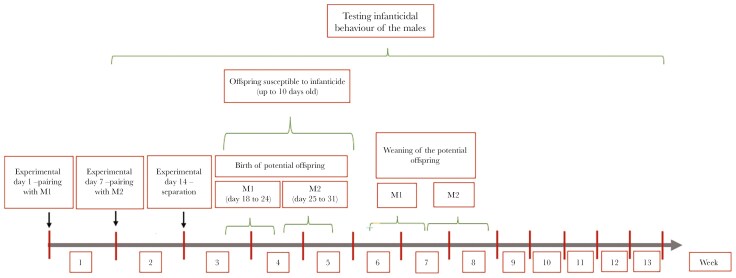
Graphical representation of the experimental setup and the important time periods (in both weeks and days) for the experiment and the study species.

Each female was paired with the first male (M1) for 7 d, after which the male was removed and replaced with the second male (M2), which stayed with the female for another 7 d. We separated all pairs on an experimental day 14, and all animals were transferred back into their single-home cages (Fig. 1.).

We assumed that offspring born until day 24 after the pairing of the female with the first male must have been sired by the first male. Births between days 25 and 29 were subjected to genetic paternity analysis (see below). As bank voles exhibit cyclic estrus, we assumed that the exchange of males after 7 d (end of the first trimester of the potential pregnancy) was assumed to allow females to get into estrus and breed with the first male but also undergo a pregnancy termination and breed with the second male (pregnancy replacement). We also considered the possibility that females were not breeding with M1 at all but only bred with M2.

It might be argued that late births in our treatments could be a general artifact of the particular experimental design, further, that by offering males sequentially, we are potentially adding opportunities for a female to conceive, which could falsely be interpreted as pregnancy replacements. We had addressed this issue in an earlier experiment in a large cohort of caged bank vole pairs (N = 202, Eccard et al. 2017), comparing overall pregnancy rates and late litter rates of females where we exchanged the breeding male, to pregnancy rates of females where the breeding male stayed with the female, otherwise using the exact same experimental setup as in this study (same pairing interval, cage size, and species). Back then we could show, that by the exchange of the breeding male the proportion of pregnancies conceived was not altered significantly (76% and 67%), while the proportion of late litters was increased (35% compared to 13%, Eccard et al. 2017). If we assume the same high pregnancy rate after the first week with the breeding male in both treatments, an increase of late births after replacement of the breeding male indicates that a proportion of females replaced the original litter. We therefore analyzed the overall pregnancy rate also in this study.

Infanticidal tendency was tested after males were returned to their home cages, therefore there is a difference in the number of infanticidal tendency sequences (infanticidal (I) or non-infanticidal (N) male). We had 4 possible infanticide tendency sequences (first male—second male): I-I (17), I-N (13), N-I (9), and N-N (32).

The males presented to the females could have been either related (a brother) or unrelated. We investigated the importance of relatedness between males and females in the pairing in the preliminary analysis, however the relatedness factor did not improve the model fit and was therefore excluded from further analysis.

### Infanticidal behavior testing

We used the testing procedure adapted from Vihervaara et al. 2010, where the male encounters a tube with a protected, unrelated pup(s) to estimate infanticidal tendencies (Vihervaara et al. 2010). For each trial, two pups unrelated to the tested male were placed in a double-walled tube (5 cm in length, 1.5 cm in diameter, made of plastic wire mesh, mesh size 1.5 mm) with a small amount of bedding from their nest. Both ends of the tube were closed so that the pups could not be harmed. The tube was placed into a corner of the testing arena (60 cm x 40 cm x 30 cm container), and the male was placed in the opposite corner without direct handling from the observer. After each trial, the arena was cleaned with 70% ethanol to remove the odors of the previously tested male and was air-dried before being reused. All pups used in the infanticide trials were 2 to 5 d old. We used the same pups repeatedly for no longer than 30 min, after which they were returned to their mother’s nest. There was no negative survival effect on repeatedly used pups.

The test started when the male was released into the arena, and we observed its behavior for 5 min. Bank voles usually start attacking the pups shortly after encountering them (Ylönen 1997), therefore a 5-min period was considered sufficient to distinguish a male’s behavior as infanticidal or non-infanticidal. To test the repeatability of infanticidal behavior. (Korpela et al. 2010; Vihervaara et al. 2010), one-third of the males underwent the test twice We classified their behavior as indicated in Vihervaara et al. 2010:

A) *Infanticidal behavior* was categorized as “aggressive” if the male approached the tube and showed aggressive behavior (biting and rolling of the tube). If the male displayed such behavior the trial was stopped, and the male was removed from the container and classified as “infanticidal.”
B) *Non-infanticidal behavior*. The male was categorized as “non-infanticidal” if he ignored the pup in the protective tube or approached it non-aggressively during the first 5 min. After the 5-min period had ended, the male was removed from the container and classified as “non-infanticidal.”

In the experiment, most males were tested up to 4 wk (28 d) after being paired (range: 2 to 13 wk; median = 4 wk) with the first female. This period would resemble the time after which a possible offspring of the male would be born (see Fig. 1.) and males’ infanticidal tendencies would temporarily disappear (Elwood 1977; Labov 1980; Cicirello and Wolff 1990; Vihervaara et al. 2010), hence males could not harm their offspring. When the offspring is more than 10 d old (ie 4 to 5 wk after the mating) males should regain their infanticidal tendencies since their own young would be mobile enough that they can escape the attack (Ylönen 1997; Horne and Ylönen 2002; Korpela et al. 2010).

Additionally, 20 males were retested in the spring 2024 to investigate the repeatability of infanticidal tendencies over time. Moreover, we wanted to assess if males exhibit the same type of behavior (aggressive or non-aggressive) when tested against an empty tube with only bedding (as a novel object exploration) compared to those with bedding and pups. Therefore, another 20 males were tested in an empty tube test and their behavior was scored as described for the infanticide test.

### Male quality assessment

Male quality was assessed in paired trials using urine-marking (UM) behavior, a commonly used method to classify dominance relationships in rodents (Desjardins et al. 1973), and specifically in bank voles (Rozenfeld et al. 1987; Horne and Ylönen 1996). While low-quality males only leave a few concentrated urine spots, high-quality males extensively mark large areas with thin, squiggly shaped traces (Rozenfeld and Rasmont 1991; Horne and Ylönen 1996), which are used to quantify the urine marking value (UMV).

Males were tested three times against random male opponents in short rounds (2 h). Tests were conducted in two connected arenas (60 cm x 42 cm x 20 cm) divided by a 42 cm long plastic wall with 5 mm holes. During the trials, males were not able to interact, but they could still detect each other using olfactory cues, which for profoundly olfactory communicators as bank voles, would be sufficient. We used a significantly repeatable urine marking style (traces or puddles) to categorize males as high or low-quality (GLMM-based intra-class correlation for binomial data: R = 0.665, CI95% = [0.341, 1.186], p = 0.001).

It has been shown that urine marking behavior is independent of the opponents’ marking behavior and identity, and the specific style (traces of puddles), as well as high or low urine marking values, are repeatable (Horne and Ylönen 1996; Klemme et al. 2006; Korpela et al. 2010; Erixon et al. 2024). Further, males with higher UMV were chosen over those with lower UMV in females’ first choices during simultaneous choice trials (Horne 1998) as well by females in sequential mate choice trials involving delayed reproduction (Vodjerek et al. 2024) indicating that these social odors signal male dominance and quality to the female. Consequently, we treated urine marking behavior as an indicator of intrinsic male quality rather than a rank relative to the opponent.

### Paternity analysis

We challenged our paternity assignment for the litters born in the second peak (days 25 to 29) using genetic paternity analyses. Tissue samples were collected from parents and litters by a small ear biopsy. DNA extraction and microsatellite amplification were done according to the protocol of Gockel et al. (1997) for up to seven microsatellite loci MSCg-4, MSCg-7, MSCg-9, MSCg-15, MSCg-18, MSCg-24 and MAR 1 (Gockel et al. 1997; Gerlach and Musolf 2000). Parentage was assigned with the software COLONY 2.0.7.0 (Jones and Wang 2010), within each family separately. We assigned paternity to 23 families using genetic parentage analyses and confirmed all of our assignments. Multiple males sired none of the litters (see also (Vodjerek et al. 2024)).

### Statistical analysis and sample sizes

All analyses were carried out in R Studio (version 4.3.3; R Studio Team 2024). First, we tested whether males’ infanticidal behavior was changing over time relative to the onset of pairing (males tested up to 4 wk: N = 38, males tested after 4 wk: N = 23) by using Generalized Linear Model (GLM) for binomial data and the function “glm” from the R package “car” (Fox and Weisberg 2011). As the predictor variable we used male infanticidal category (infanticidal/non-infanticidal), and as a fixed factor the “week from the first pairing” (how many weeks has passed between infanticidal behavior testing and pairing with the female, see Fig. 1.)

To establish if infanticidal tendencies were a repeatable trait we used GLMM-based intra-class correlation using the “rpt” function from the R package “rptR” (Stoffel et al. 2017). The testing occasion/round (first or second test) did not have a significant effect on the results; therefore we could use the simpler, intercept-only, model. The infanticidal tendency category (infanticidal or non-infanticidal) was used as a response variable. Further, the same model and analysis were used to explore whether there was any association between male behavior when tested in an empty tube test (exploration/playful behavior) and an infanticide test.

First, we tested whether the probability of a litter to be born differed between four infanticidal tendency sequences using a Generalized Linear Mixed Model (GLMM) for binomial data and the function “glmer” of the R package “lme4” (Bates et al. 2015). As a predictor variable we used “female pregnant” (yes/no). As a fixed factor we used infanticidal tendency sequence (4 levels of sequence by infanticidal tendency combination: I-I, I-N, N-I, and N-N), and female identity (N=54) was used as a random (intercept) factor as 34 females we included in the pairing two times. Further, to explore if the probability of a litter to be born late (N = 71) differed between different infanticidal tendency sequences we used a Generalized Linear Mixed Model (GLMM) for binomial data, using the function “glmer” of the R package “lme4” (Bates et al. 2015). Each sampling unit is a litter. We included the infanticidal tendency sequence as a fixed effect (4 levels: I-I, I-N, N-I, and N-N), and female identity (N=46) was used as a random (intercept) factor since 25 females were included in the experiment two times. We also investigated infanticidal males’ position in the sequence (2 levels: I-N and N-I) had an effect on timing of birth (early or late litter) using GLMM (for binomial data) with female identity as random (intercept) factor.

In addition, to estimate the effects of individual males’ infanticidal tendencies on its probability to reproduce we used GLMM for binomial data (N = 142), with the response being the males’ reproduction (yes/no) in a pairing. In the model, we included the infanticidal tendency of a male, sequence (in the pairing) and their interaction as fixed effects. To control for their potential effects, we included male quality and female parity as further fixed effects. As random (intercept) effects, we included male identity (N = 61), and female identity (N = 46), because most females were in the experiment twice (with different male dyads) and males were used several times in different experimental male dyads. To capture the sequence of male properties presented to the female, we included the unique triplet (Female - M1 - M2) to the model as a further random (intercept) factor. Post-hoc comparisons to further explore this significant interaction term were carried out using the package “lsmeans” (Lenth 2016).

Other statistical analyses and packages used to obtain them: WALD chi-square test (“car” (Fox and Weisberg 2011)), R^2^ values (‘MuMIn’ (Bartoń 2024)), and residual diagnostics (“DHARMa” (Hartig 2018)).

## Results

### Male infanticidal behavior

Of 61 tested male bank voles, 23 (38%) were categorized as infanticidal, and 38 (62%) as non-infanticidal. Infanticidal males were not heavier (26.3 ± 4.9 g, mean ± SD) than non-infanticidal males (25.5 ± 3.1 g, mean ± SD; t = 0.71, df = 58.9, p = 0.481), and there was no association between male quality and the infanticidal category (χ2 = 1.66, df = 1, p = 0.198). Infanticidal behavior was not changing over time relative to the onset of pairing (GLM binomial: χ2 = 5.21, df = 7, p = 0.634; see Fig. 1.). Further, male infanticidal tendencies were not repeatable over long periods of life (males tested in the season when they were paired/mated with a female and in a season when no pairing/mating occurred; GLMM-based intra-class correlation: R = 0.087, CI95% = [0, 0.587], p = 0.329). There was also no association between the behavior (aggressive/non-aggressive) in the empty tube test and the infanticide test (GLMM-based intra-class correlation: R = 0.015, SE = 0.117, CI95% = [0, 0.446], p = 0.466).

### Reproduction

Out of 61 males, 13 did not sire a litter during the experiment (21%, in 20% of sequences), however, siring a litter was not significantly associated with the infanticidal tendencies of these males (χ2 = 1.25, df = 1, p = 0.264) or their quality (χ2 = 1.26, df = 1, p = 0.261). Since we could not distinguish whether these males did not sire offspring due to the quality of their dyadic opponent, or whether the individuals could have been infertile, we conducted additional analyses with a reduced dataset excluding them (13 dyads). Both analyses yielded the same results, therefore, we report the results of the full dataset.

Out of 88 pairings made, 71 litters (81%) were obtained. Non-breeding females were distributed evenly among different infanticidal tendency sequences: 2/19 (11%) unsuccessful matings in the sequence I-I, 3/16 (19%) in sequence I-N, 4/13 (31%) in sequence N-I and 8/40 (20%) in sequence N-N (GLMM: χ2 = 1.46, df = 3, p = 0.691).

**Fig. 2.**
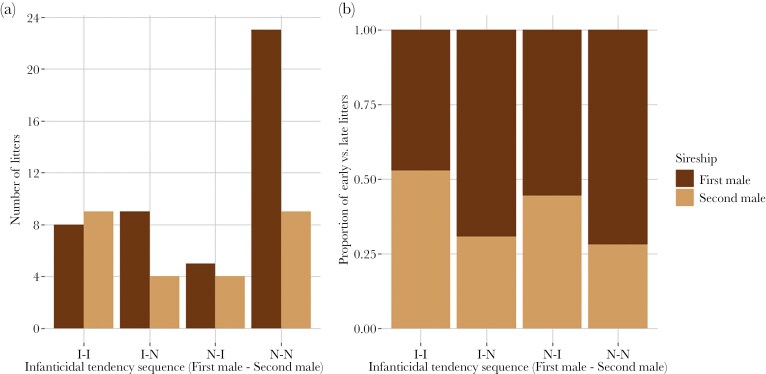
Bank vole litters that were sired by the first and second males in a sequential pairing experiment, where each male stayed with the female for one week. Males were categorized as infanticidal (I), or noninfanticidal (N), and delivery of litters sired by the second male probably included a pregnancy replacement, indicating infanticide avoidance in the case where the second male was infanticidal (I-I and N-I). A) The number, and B) the proportion of early and late litters, per infanticidal tendency sequence.

We tested whether the number of late litters differed between four infanticidal tendencies sequences (N = 71). If none of the males in the pairing was infanticidal, the majority of litters (72%) was sired be the first male. The number of late litters increased notably in response to the second male being infanticidal (50%; sequences I-I and N-I) compared to the second male being non-infanticidal (29%, sequences I-N and N-N; χ2 = 2.77, df = 1, p = 0.09, Fig. 2.). Further, if one of the males was infanticidal (sequences I-N and N-I), 59% of the litters were sired by this male (GLM for binomial data, N = 22, χ2 = 0.43, df = 1, p = 0.513) and if both males were infanticidal, 52% of the litters were sired by the second male. However, no significant difference between the 4 infanticidal tendency sequences could be detected (χ2 = 2.89, df = 3, p = 0.409, Fig. 2.).

**Fig. 3.**
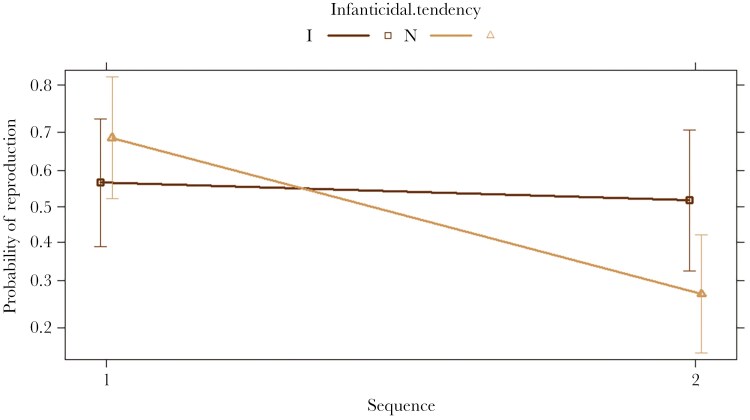
Predicted probability for males to reproduce (Table 1.) as a function of pairing sequence (1st or 2nd in the pairing) and infanticidal tendency (male infanticidal (I) and non-infanticidal (N)).

Additionally, we tested the probability of reproduction for 142 males in 71 male dyads. We found a significant interaction between the male sequence and infanticidal tendency (χ2 = 4.78, df = 1, p = 0.028, Fig. 3., Table 1.). More specifically, the probability of reproducing for non-infanticidal males was significantly lower if they were the second in the pairing (Z ratio = 3.54, p = 0.002; Fig. 3.) and the probability of reproducing tended to be higher for infanticidal males that were first in the pairing compared to the non-infanticidal males being second in the pairing (Z ratio = 2.49, p = 0.062). We did not find any significant effects of male quality or female parity (all p > 0.10; Table 1.)

**Table 1.** **Male based model** ^*^. Results of the model (GLMM for binomial data) regarding the effect of individual males’ infanticidal tendencies on reproduction with random (intercept) factors being male identity (N = 61), female identity (N = 46) and unique triplet (female - M1 - M2; N = 71).

Model	Response	Explanatory variable	Est	Std Error	CL_lower_	CL_upper_	z	p
Male based model (GLMM) N = 142, R^2^m = 0.13	Reproduction (yes/no)	(Intercept) Infanticidal tendency I ~ N ** Sequence 2 ~ 1** Quality Low ~ High Female parity P ~ N Infanticide * Sequence	0.784 -0.509 **-1.777** 0.041 -0.098 **1.573**	0.415 0.503 **0.478** 0.363 0.362 **0.719**	-0.006 -1.690 **-3.350** -0.757 -0.944 **0.179**	1.858 0.560 **-0.943** 0.865 0.768 **3.513**	1.876 -0.994 **-3.544** 0.110 -0.270 **2.186**	0.061. 0.321 **<0.001** 0.912 0.787 **0.029**

^*^From the model, we report estimates including their standard errors and 95% confidence limits, z values, and p-values. The first-factor level is used as a reference level. Explanatory variables with significant effects are shown in bold.

## Discussion

Infanticide by non-parental males was one of the earliest recognized forms of sexual conflict. In our study, we found that the proportion of late births increased significantly, by 21%, if the second male encountered was infanticidal, compared to the second male being non-infanticidal. Further, if one of the males encountered by the female was infanticidal, the majority of litters were sired by this male (59%). For a short-lived mammal, such as a bank vole, timing is of the essence when it comes to reproduction. It is important to reproduce as fast as possible before the population increases in early summer, and the habitat gets saturated by individuals. Therefore, breeding early will ensure that individuals’ genes are transferred to the next generation, and consequently, individuals’ fitness is increased. However, females might have to choose if they are willing to risk that their “early” offspring are killed by an invading male, or they might delay the birth of their offspring to avoid infanticide. In our study, we showed that a large portion of females chose to delay the birth rather than risk infanticide by the new male encountered showing, to a certain extent, the predictive power of the infanticidal status of males on the timing of pregnancies. Finally, the probability of reproduction for both infanticidal and non-infanticidal males depended on their position in the sequence, specifically non-infanticidal males had a significantly higher probability of reproduction if they were first in the sequence, compared to being second in the sequence. Similarly, infanticidal males had a higher probability of reproduction when first in the sequence, compared to non-infanticidal males being second in the sequence. This shows us the order in which certain males are encountered, together with their infanticidal tendencies, would consequently decide which male would sire the offspring. As bank voles are a promiscuous species, not mating with the first male was highly unlikely, especially when considering a very high pregnancy rate of 81% we had in this experiment. With no differences in pregnancy rate between treatments, but differences in late litter rates, we can presume that the late litters were indeed a result of pregnancy replacement rather than a result of mating only with the second male (see also Eccard et al. 2017).

One of the more prominent questions that arises when one mentions infanticide is whether females are able to recognize infanticidal males. What we know is that olfactory communication is the most prominent in rodents (Schultz and Tapp 1973; Kelliher 2007) and thereby in bank voles. As “urine and fecal marks convey olfactory signals characteristic of the species, the sex and the hierarchical background of the animal that deposited them,” odors can act as an indicator of nutritional status of condition of potential mates, age, genotype etc. (Desjardins et al. 1973; Hoffmeyer 1982; Yamazaki et al. 1983; Rozenfeld and Rasmont 1991; Horne and Ylönen 1996; Penn and Potts 1998; Ferkin 2018). Even though infanticidal tendencies are not related to the urine marking value (UMV; Horne and Ylönen 2002; Korpela et al. 2010)), other behavioral properties (such as aggression) are (Erixon et al. 2024). As a result, we assume and speculate, that females may directly assess these different behaviors and odors, and therefore indirectly gain information on males’ infanticidal tendencies. Due to the higher proportion of late litters sired by infanticidal males (50%) in all sequences, and higher proportion of litters sired by the infanticidal male in sequences where one of the males was infanticidal (59%), we can safely assume that females can assess males’ infanticidal tendencies.

In order to commit infanticide, males need to be able to find nests with the pups, and we have tested whether the behavior males show (ie aggressive behavior) towards an empty tube and a tube with pups will be the same or different. We know from previous research on rodents, bank voles included, that they can hear ultrasound, as they use it to communicate with each other, and this type of communication is specifically important in mother-offspring interaction (Sewell 1970; Kober et al. 2007; Matrosova et al. 2007). Male voles are therefore able to listen in on mother-offspring communication and use it to find the nest and commit infanticide. As in our infanticide test pups were taken from the mother and the nest for around 30 min, they probably produced ultrasonic vocalization in order to call out for their mother. This might have attracted infanticidal males, as it was shown that infanticidal males are more attracted to nest boxes with nest cues (Ylönen et al. 2017). Alternatively, as bank vole pups are probably releasing distress signals in the absence of their mother, this might have attracted some males who would try to free them from the tube. However, this scenario is very unlikely since bank vole males are not exhibiting any parental care (Ylönen et al. 2017), therefore, distress signals (separation calls) emitted by the pups probably would not affect their behavior in a way that they would have shown care. Males showed different behavior during the infanticidal test, ie different forms of tube exploration (just sniffing, biting, rolling etc.) we wanted to make sure that this behavior is not related to playful/exploratory behavior since they are encountering the tube and the offspring for the first time. Therefore, we tested males’ behavior towards a tube with bedding and compared it to their behavior in the infanticide test (a tube with bedding and pups). We found no association between the behavior in an empty tube test and the infanticide test. Accordingly, we concluded that the aggressive behavior males were showing toward the tube with the pups was indeed associated with their infanticidal tendencies towards the pups.

It has been shown that infanticide is inhibited when mated males’ offspring are born (Elwood 1977; Labov 1980; Cicirello and Wolff 1990). Two primary hypotheses have been proposed to explain the suppression of infanticide in males: one suggests that copulation alone would inhibit infanticide, while the other poses that previous association with the mother is crucial for inhibition to take place. These hypotheses were tested in bank voles as well but have not met the same conclusions (Vihervaara et al. 2010). In this study, the authors propose that multiple cues acting simultaneously would be needed to inhibit infanticide, such as several matings of a female with the same male, but also familiarization of a male with the site, the female territory within his own home range, if not with the female itself (Vihervaara et al. 2010). In our experiment, we did not find male infanticidal behavior to be repeatable over long periods. Therefore, we suggest that infanticidal behavior should not be considered an individual, behavioral trait, but rather a behavior dependent on the reproductive context.

Females have evolved numerous counter-strategies to reduce the risk of infanticide (Agrell et al. 1998; Ebensperger 1998). Pregnancy block or ‘Bruce effect’ has been suggested as one of the mechanisms to avoid infanticide, especially since it may prevent investing in infants that may be killed by the new, invading male (Hrdy 1979; Schwagmeyer 1979; Labov 1980, 1981; Agrell et al. 1998; Ebensperger 1998). This hypothesis predicts that there should be a direct correlation between the rate of pregnancy block and the risk of infanticide and that females who block their pregnancies should wean more offspring (Ebensperger 1998). In the present study, we tested infanticide avoidance in 4 different infanticidal tendency sequences where 71 litters were obtained, and we found no overall significant difference in the litter production between the sequences. The percentage of late litters differed significantly in the sequences where the infanticidal male was encountered as the second (I-I and N-I, 50%), compared to 29% of late litters if the second male encountered was not infanticidal (I-N and N-N). Further, we found a significant interaction between males’ infanticidal tendencies and the sequence in which they were presented to the females. Our results suggest that there is a higher probability of reproduction if the non-infanticidal male is the first one in the pairing. Consequently, there is some predictive power of males’ infanticidal tendencies on the timing of the birth. Another strategy that females might use is to mate with the dominant male, since in many small mammal species such males are more infanticidal against non-sired offspring (Huck et al. 1982; Vom Saal and Howard 1982), and are more likely to attack and fend-off the intruders than subordinate males. Thus, females would gain from selecting dominant males, who would pose a greater threat to their offspring if they were not the sires. Further, previous research on mice showed that a higher proportion of high-quality (dominant) males were infanticidal, in contrast to low-quality (subordinate) males (Huck 1982), yet in our study no connection was found between male infanticidal tendency and male quality, which is in line with previous findings in bank voles (Horne and Ylönen 2002; Korpela et al. 2010). The tendency to choose dominant males is widespread throughout the animal kingdom as it is an indication of good genotype, phenotype and resource access, and associating with dominant males might reduce the probability of infanticide. In free mate choice experiments where males were not able to leave their compartments, and females would visit them, dominant males had a higher paternity success (Klemme et al. 2006). Even though we showed a preference for high quality males in an earlier experiment (Vodjerek et al 2024) where the pregnancy replacement rates were higher if the second male encountered was of high-quality, in the present experiment we could not find the same preference. Possibly the effect of quality might be weaker, or masked, in this experiment by controlling for infanticidal tendency and sequence of infanticidal males.

## Conclusion

In conclusion, we found that the number of late litters, the indicator of pregnancy replacement, was higher if the second male encountered was infanticidal. Further, the probability of reproducing in both infanticidal and non-infanticidal males depended on whether they were encountered first or second by the female. We found no association between infanticidal tendencies and male quality, and male infanticidal tendencies were not repeatable over long periods. For individuals of short-lived species, often with annual density fluctuations, it is important to reproduce as early as possible to contribute to the future population. Thus, by reproducing late, females lose essential time and possibly a major proportion of fitness. Meanwhile, in our experiment, females chose to delay the pregnancy in order to avoid possible infanticide by males. Therefore, with this experiment, we confirmed that the infanticidal status of the males and the sequence in which the males were presented to the females delayed the timing of the births and influenced the males’ probability of reproducing. These results possibly indicate that there is a strong selection on females to use pregnancy replacement as a counterstrategy to avoid infanticide by males, even at the cost of delaying reproduction.

## Data Availability

Analyses reported in this article can be reproduced using the data provided by Vodjerek (2025).

## References

[CIT0001] Agrell J , Wolff JO, Ylönen H, Ylonen H. 1998. Counter-strategies to infanticide in mammals: costs and consequences. Oikos. 83:507. https://doi.org/10.2307/3546678

[CIT0002] Andersson CB , Gustafsson TO. 1982. Effect of limited and complete mating on ovaries and adrenals in bank voles, *Clethrionomys glareolus*. Reproduction. 64:431–435. https://doi.org/10.1530/jrf.0.0640431 7040648

[CIT0003] Arnqvist G. 2004. Sexual conflict and sexual selection: lost in the chase. Evolution. 58:1383–1388. https://doi.org/10.1554/03-449 15266986

[CIT0004] Bartoń K. 2024. MuMIn: Multi-Model Inference. R Package Version 1.48.4. https://cran.r-project.org/web/packages/MuMIn/index.html

[CIT0005] Bartoš L , Putman R, Pluháček J, Dušek A, Bartošová J. 2022. Bruce effect, pregnancy block and disruption or feticide: proposal of a new term “effect of nonsire male’s presence.” Anim Behav. 187:117–119. https://doi.org/10.1016/j.anbehav.2022.03.008

[CIT0006] Bates D , Mächler M, Bolker B, Walker S. 2015. Fitting linear mixed-effects models using lme4. J Stat Softw. 67:1–48. https://doi.org/10.18637/jss.v067.i01

[CIT0007] Blumstein DT. 2000. The evolution of infanticide in rodents: a comparative analysis. In: Van Schaik CP, Janson, CH, editors. Infanticide by males and its implications. Cambridge University Press. p. 178–197.

[CIT0008] Breedveld MC , Folkertsma R, Eccard JA. 2019. Rodent mothers increase vigilance behaviour when facing infanticide risk. Sci Rep. 9:12054. https://doi.org/10.1038/s41598-019-48459-9 31427633 PMC6700316

[CIT0009] Bruce HM. 1959. An exteroceptive block to pregnancy in the mouse. Nature. 184:105–105. https://doi.org/10.1038/184105a0 13805128

[CIT0010] Bruce HM. 1960. A block to pregnancy in the mouse caused by proximity of strange male. J Reprod Fertil. 1:96–103. https://doi.org/10.1530/jrf.0.0010096 13805127

[CIT0011] Bujalska G. 1990. Social system of the bank vole, *Clethrionomys glareolus.* In: Tamarin RH, Ostfeld RS, Pugh SR, Bujalska G, editors. Social systems and population cycles in voles. Birkhäuser Basel. p. 155–167. http://link.springer.com/10.1007/978-3-0348-6416-9_15

[CIT0012] Calhoun JB. 1962. Population density and social pathology. Sci Am. 206:139–148. https://doi.org/10.1038/scientificamerican0262-139 13875732

[CIT0013] Chapman M , Hausfater G. 1979. The reproductive consequences of infanticide in langurs: a mathematical model. Behav Ecol Sociobiol. 5:227–240. https://doi.org/10.1007/bf00293672

[CIT0014] Cicirello DM , Wolff JO. 1990. The effects of mating on infanticide and pup discrimination in white-footed mice. Behav Ecol Sociobiol. 26. https://doi.org/10.1007/BF00178320

[CIT0015] Clutton-Brock TH , Parker GA. 1992. Potential reproductive rates and the operation of sexual selection. Q Rev Biol. 67:437–456. https://doi.org/10.1086/417793

[CIT0016] Desjardins C , Maruniak JA, Bronson FH. 1973. Social rank in house mice: differentiation revealed by ultraviolet visualization of urinary marking patterns. Science. 182:939–941. https://doi.org/10.1126/science.182.4115.939 4745598

[CIT0017] Dewsbury DA. 1990. Modes of estrus induction as a factor in studies of the reproductive behavior of rodents. Neurosci Biobehav Rev. 14:147–155. https://doi.org/10.1016/s0149-7634(05)80215-5 2190114

[CIT0018] Ebensperger LA. 1998. Strategies and counterstrategies to infanticide in mammals. Biol Rev. 73:321–346. https://doi.org/10.1017/s0006323198005209

[CIT0019] Ebensperger LA , Blumstein DT. 2007. Nonparental infanticide. In: Wolff JO, Sherman PW, editors. Rodent societies. University of Chicago Press. p. 267–279.

[CIT0020] Eccard JA , Dammhahn M, Ylönen H. 2017. The Bruce effect revisited: is pregnancy termination in female rodents an adaptation to ensure breeding success after male turnover in low densities? Oecologia. 185:81–94. https://doi.org/10.1007/s00442-017-3904-6 28791488 PMC5596041

[CIT0021] Elgar MA , Crespi BJ, editors. 1992. Cannibalism: ecology and evolution among diverse taxa. Oxford University Press.

[CIT0022] Elwood RW. 1977. Changes in the responses of male and female gerbils (*Meriones unguiculatus*) towards test pups during the pregnancy of the female. Anim Behav. 25:46–51. https://doi.org/10.1016/0003-3472(77)90066-5

[CIT0023] Elwood RW , Kennedy HF. 1990. The relationship between infanticide and pregnancy block in mice. Behav Neural Biol. 53:277–283. https://doi.org/10.1016/0163-1047(90)90526-c 2331236

[CIT0024] Erixon F , Eccard JA, Huneke R, Dammhahn M. 2024. A behavioral syndrome of competitiveness in a non-social rodent. Behav Ecol Sociobiol. 78:98. https://doi.org/10.1007/s00265-024-03510-2

[CIT0025] Ferkin M. 2018. Odor communication and mate choice in rodents. Biology. 7:13. https://doi.org/10.3390/biology7010013 29370074 PMC5872039

[CIT0026] Fox J , Weisberg S. 2011. An R companion to applied regression. Second Edition. SAGE Publications.

[CIT0027] Gerlach G , Musolf K. 2000. Fragmentation of landscape as a cause for genetic subdivision in bank voles. Conserv Biol. 14:1066–1074. https://doi.org/10.1046/j.1523-1739.2000.98519.x

[CIT0028] Glass GE , Holt RD, Slade NA. 1985. Infanticide as an evolutionarily stable strategy. Anim Behav. 33:384–391. https://doi.org/10.1016/s0003-3472(85)80062-2

[CIT0029] Gockel J , et al 1997. Isolation and characterization of microsatellite loci from *Apodemus flavicollis* (rodentia, muridae) and *Clethrionomys glareolus* (rodentia, cricetidae). Mol Ecol. 6:597–599. https://doi.org/10.1046/j.1365-294x.1997.00222.x 9200832

[CIT0030] Gustafsson T , Andersson B, Westlin L. 1980. Reproduction in a laboratory colony of bank vole, *Clethrionomys glareolus*. Can J Zool. 58:1016–1021. https://doi.org/10.1139/z80-142 7000324

[CIT0031] Harestad A , Davis H. 1996. Cannibalism by black bears in the Nimpkish Valley, British Columbia. Northwest Sci. 70:88–92.

[CIT0032] Hartig F. 2018. DHARMa: residual diagnostics for hierarchical (multi-level/mixed) regression models. R package version 0.2.0. https://CRAN.R-project.org/package=DHARMa

[CIT0033] Hausfater G , Hrdy SB. 1984. Infanticide: comparative and evolutionary perspectives. 1st ed. Routledge.

[CIT0034] Heske EJ. 1987. Pregnancy interruption by strange males in the California vole. J Mammal. 68:406–410. https://doi.org/10.2307/1381485

[CIT0035] Heske EJ , Nelson RJ. 1984. Pregnancy interruption in *Microtus ochrogaster*: laboratory artifact or field phenomenon? Biol Reprod. 31:97–103. https://doi.org/10.1095/biolreprod31.1.97 6380603

[CIT0036] Hiraiwa-Hasegawa M. 1988. Adaptive significance of infanticide in primates. Trends Ecol Evol. 3:102–105. https://doi.org/10.1016/0169-5347(88)90116-4 21227158

[CIT0037] Hoffmeyer I. 1982. Responses of female bank voles (*Clethrionomys glareolus*) to dominant vs subordinate conspecific males and to urine odors from dominant vs subordinate males. Behav Neural Biol. 36:178–188. https://doi.org/10.1016/s0163-1047(82)90167-4 6763863

[CIT0038] Hoogland JL. 1985. Infanticide in prairie dogs: lactating females kill offspring of close kin. Science. 230:1037–1040. https://doi.org/10.1126/science.230.4729.1037 17814930

[CIT0039] Horne TJ. 1998. Evolution of female choice in the bank vole [Ph.D. Thesis]. University of Jyväskylä.

[CIT0040] Horne TJ , Ylönen H. 1996. Female bank voles (*Clethrionomys glareolus*) prefer dominant males; but what if there is no choice? Behav Ecol Sociobiol. 38:401–405. https://doi.org/10.1007/s002650050257

[CIT0041] Horne TJ , Ylönen H. 2002. Infanticide and effectiveness of pup protection in bank voles: does the mother recognise a killer? Acta Ethologica. 4:97–101. https://doi.org/10.1007/s10211-001-0055-9

[CIT0042] Hrdy SB. 1979. Infanticide among animals: A review, classification, and examination of the implications for the reproductive strategies of females. Ethol Sociobiol. 1:13–40. https://doi.org/10.1016/0162-3095(79)90004-9

[CIT0043] Huck UW. 1982. Pregnancy block in laboratory mice as a function of male social status. J Reprod Fertil. 66:181–184. https://doi.org/10.1530/jrf.0.0660181 7120182

[CIT0044] Huck UW. 1984. Infanticide and the evolution of pregnancy block in rodents. In: Hausfater G, Hrdy SB, editors. Infanticide: comparative and evolutionary perspectives. ldine Publishing Company. p. 349–365.

[CIT0045] Huck WU , Soltis RL, Coopersmith CB. 1982. Infanticide in male laboratory mice: Effects of social status, prior sexual experience, and basis for discrimination between related and unrelated young. Anim Behav. 30:1158–1165. https://doi.org/10.1016/S0003-3472(82)80206-6

[CIT0046] Jones OR , Wang J. 2010. COLONY: a program for parentage and sibship inference from multilocus genotype data. Mol Ecol Resour. 10:551–555. https://doi.org/10.1111/j.1755-0998.2009.02787.x 21565056

[CIT0047] Kelliher KR. 2007. The combined role of the main olfactory and vomeronasal systems in social communication in mammals. Horm Behav. 52:561–570. https://doi.org/10.1016/j.yhbeh.2007.08.012 17959176 PMC2756530

[CIT0048] Klemme I , Eccard JA, Ylönen H. 2006. Do female bank voles (*Clethrionomys glareolus*) mate multiply to improve on previous mates? Behav Ecol Sociobiol. 60:415–421. https://doi.org/10.1007/s00265-006-0181-5

[CIT0049] Klemme I , Eccard JA, Ylönen H. 2007. Why do female bank voles, *Clethrionomys glareolus*, mate multiply? Anim Behav. 73:623–628. https://doi.org/10.1016/j.anbehav.2006.07.010

[CIT0050] Klemme I , Kataja-aho S, Eccard JA, Ylönen H. 2011. Variable mode of estrus affects female decision for multiple mating. Behav Ecol. 23:361–367. https://doi.org/10.1093/beheco/arr193

[CIT0051] Klemme I , Ylönen H. 2010. Polyandry enhances offspring survival in an infanticidal species. Biol Lett. 6:24–26. https://doi.org/10.1098/rsbl.2009.0500 19675002 PMC2817239

[CIT0052] Kober M , Trillmich F, Naguib M. 2007. Vocal mother–pup communication in guinea pigs: effects of call familiarity and female reproductive state. Anim Behav. 73:917–925. https://doi.org/10.1016/j.anbehav.2006.06.020

[CIT0053] Korpela K , Sundell J, Ylönen H. 2010. Density dependence of infanticide and recognition of pup sex in male bank voles. Behaviour. 147:871–881. https://doi.org/10.1163/000579510x495780

[CIT0054] Labov JB. 1980. Factors influencing infanticidal behavior in wild male house mice (*Mus musculus*). Behav Ecol Sociobiol. 6:297–303. https://doi.org/10.1007/bf00292772

[CIT0055] Labov JB. 1981. Pregnancy blocking in rodents: adaptive advantages for females. Am Naturalist. 118:361–371. https://doi.org/10.1086/283828

[CIT0056] Lenth RV. 2016. Least-Squares Means: the *R* package lsmeans. J Stat Soft. 69. https://doi.org/10.18637/jss.v069.i01

[CIT0057] Lukas D , Huchard E. 2014. The evolution of infanticide by males in mammalian societies. Science. 346:841–844. https://doi.org/10.1126/science.1257226 25395534

[CIT0058] Mappes T , et al 2012. Advantage of rare infanticide strategies in an invasion experiment of behavioural polymorphism. Nat Commun. 3:611. https://doi.org/10.1038/ncomms1613 22215086 PMC3272565

[CIT0059] Marashi V , Rülicke T. 2012. The Bruce effect in Norway rats. Biol Reprod. 86:1–5. https://doi.org/10.1095/biolreprod.111.093104 21957190

[CIT0060] Matrosova VA , Volodin IA, Volodina EV, Babitsky AF. 2007. Pups crying bass: vocal adaptation for avoidance of age-dependent predation risk in ground squirrels? Behav Ecol Sociobiol. 62:181–191. https://doi.org/10.1007/s00265-007-0452-9

[CIT0061] McCarthy MM , Vom Saal FS. 1986. Inhibition of infanticide after mating by wild male house mice. Physiol Behavior. 36:203–209. https://doi.org/10.1016/0031-9384(86)90004-1 3960991

[CIT0062] Mennella JA , Moltz H. 1988. Infanticide in rats: male strategy and female counter-strategy. Physiol Behavior. 42:19–28. https://doi.org/10.1016/0031-9384(88)90254-5 3387474

[CIT0063] Milligan SR. 1976. Pregnancy blocking in the vole, *Microtus agrestis*. Reproduction. 46:97–100. https://doi.org/10.1530/jrf.0.0460097 775074

[CIT0064] Opperbeck A , Ylönen H, Klemme I. 2012. Infanticide and population growth in the bank vole (*Myodes glareolus*): the effect of male turnover and density. Ethology. 118:178–186. https://doi.org/10.1111/j.1439-0310.2011.01998.x

[CIT0065] Palanza P , Parmigiani S. 1991. Inhibition of infanticide in male Swiss mice: Behavioral polymorphism in response to multiple mediating factors. Physiol Behav. 49:797–802. https://doi.org/10.1016/0031-9384(91)90320-n 1881986

[CIT0066] Parker GA. 1979. Sexual selection and sexual conflict. In: Blum MS, Blum NA, editors. Sexual selection and reproductive competition in insects. Academic Press. p. 123–166.

[CIT0067] Parmigiani S , Vom Saal F, editors. 1994. Infanticide and parental care. 1st ed. Harwood Academic Publishers. https://www.taylorfrancis.com/books/9781134947966

[CIT0068] Penn D , Potts WK. 1998. Untrained mice discriminate MHC-determined odors. Physiol Behavior. 64:235–243. https://doi.org/10.1016/s0031-9384(98)00052-3 9748088

[CIT0069] Pierotti R. 1991. Infanticide versus adoption: an intergenerational conflict. Am Nat. 138:1140–1158. https://doi.org/10.1086/285274

[CIT0070] Poikonen T , Koskela E, Mappes T, Mills SC. 2008. Infanticide in the evolution of reproductive synchrony: effects on reproductive success. Evolution. 62:612–621. https://doi.org/10.1111/j.1558-5646.2007.00293.x 17983462

[CIT0091] Posit team (2024). RStudio: Integrated Development Environment for R. Posit Software, PBC, Boston, MA. http://www.posit.co/.

[CIT0071] Ratkiewicz M , Borkowska A. 2000. Multiple paternity in the bank vole (*Clethrionomys glareolus*): field and experimental data. Int J Mamm Biol. 65: 6–14.

[CIT0072] Roberts EK , Lu A, Bergman TJ, Beehner JC. 2012. A Bruce effect in wild geladas. Science. 335:1222–1225. https://doi.org/10.1126/science.1213600 22362878

[CIT0073] Rozenfeld FM , Boulangé EL, Rasmont R. 1987. Urine marking by male bank voles (*Clethrionomys glareolus* Schreber, 1780; Microtidae, Rodentia) in relation to their social rank. Can J Zool. 65:2594–2601. https://doi.org/10.1139/z87-393

[CIT0074] Rozenfeld FM , Rasmont R. 1991. Odour cue recognition by dominant male bank voles, *Clethrionomys glareolus*. Anim Behav. 41:839–850. https://doi.org/10.1016/s0003-3472(05)80351-3

[CIT0075] Rudran R. 1973. Adult Male Replacement in One-Male Troops of purple-faced langurs (*Presbytis senex senex*) and its effect on population structure. Folia Primatol (Basel). 19:166–192. https://doi.org/10.1159/000155537 4201908

[CIT0076] Schultz EF , Tapp JT. 1973. Olfactory control of behavior in rodents. Psychol Bull. 79:21–44. https://doi.org/10.1037/h0033817 4567728

[CIT0077] Schwagmeyer PL. 1979. The Bruce effect: an evaluation of male/female avantages. Am Nat. 114:932–938. https://doi.org/10.1086/283541

[CIT0078] Sewell GD. 1970. Ultrasonic signals from rodents. Ultrasonics. 8:26–30. https://doi.org/10.1016/0041-624x(70)90795-x 5414679

[CIT0079] Stoffel MA , Nakagawa S, Schielzeth H. 2017. rptR: repeatability estimation and variance decomposition by generalized linear mixed‐effects models. Goslee S, editor. Methods Ecol Evol. 8:1639–1644. https://doi.org/10.1111/2041-210x.12797

[CIT0080] Storey AE , Snow DT. 1990. Postimplantation pregnancy disruptions in meadow voles: relationship to variation in male sexual and aggressive behavior. Physiol Behav. 47:19–25. https://doi.org/10.1016/0031-9384(90)90037-5 2183249

[CIT0081] Trivers RL. 1972. Parental investment and sexual selection. In: Campbell B, editor. Sexual selection and the descent of man. Heinemann. p. 136–179.

[CIT0082] Vihervaara H , Sundell J, Ylönen H. 2010. Is mating alone enough to inhibit infanticide in male bank voles? Ethology. 116:888–894. https://doi.org/10.1111/j.1439-0310.2010.01806.x

[CIT0083] Vodjerek L. 2025. Risking infanticide or losing precious time? Delay of reproduction in a short-lived mammal. Behav Ecol. https://doi.org/10.5061/dryad.pg4f4qrzz PMC12096009 40416936

[CIT0084] Vodjerek L , Erixon F, Mendes Ferreira C, Fickel J, Eccard JA. 2024. The role of male quality in sequential mate choice: pregnancy replacement in small mammals? R Soc Open Sci. 11:240189. https://doi.org/10.1098/rsos.240189 39076357 PMC11285816

[CIT0085] Vom Saal FS , Howard LS. 1982. The regulation of infanticide and parental behavior: implications for reproductive success in male mice. Science. 215:1270–1272. https://doi.org/10.1126/science.7058349 7058349

[CIT0086] Yamazaki K , et al 1983. Recognition of H-2 types in relation to the blocking of pregnancy in mice. Science. 221:186–188. https://doi.org/10.1126/science.6857281 6857281

[CIT0087] Ylönen H. 1997. Infanticide in the bank vole (*Clethrionomys glareolus*): occurrence and the effect of familiarity on female infanticide. Ann Zool Fenni. 34:259–266. https://www.jstor.org/stable/23735490

[CIT0088] Ylönen H , Kasi M, Opperbeck A, Haapakoski M, Sundell J. 2017. How do infanticidal male bank voles (*Myodes glareolus*) find the nest with pups? Herberstein M, editor. Ethology. 123:105–112. https://doi.org/10.1111/eth.12579

[CIT0089] Zipple MN. 2020. When will the Bruce effect evolve? The roles of infanticide, feticide and maternal death. Anim Behav. 160:135–143. https://doi.org/10.1016/j.anbehav.2019.11.014

[CIT0090] Zipple MN , Roberts EK, Alberts SC, Beehner JC. 2021. The Bruce effect should be defined by function, not mechanism: comments on “How to escape male infanticide: mechanisms for avoiding or terminating pregnancy in mammals.”. Mammal Rev. 51:596–599. https://doi.org/10.1111/mam.12250

